# Physical activity in pregnancy: a qualitative study of the beliefs of overweight and obese pregnant women

**DOI:** 10.1186/1471-2393-10-18

**Published:** 2010-04-28

**Authors:** Zoe Weir, Judith Bush, Stephen C Robson, Catherine McParlin, Judith Rankin, Ruth Bell

**Affiliations:** 1 Institute of Health & Society, Newcastle University, Medical Sciences New Building, Richardson Road, Newcastle upon Tyne, NE2 4AX, UK; 2 Institute of Cellular Medicine, William Leech Building, Newcastle University, Newcastle upon Tyne NE2 4HH, UK; 3 Newcastle upon Tyne Hospitals NHS Foundation Trust, Royal Victoria Infirmary, Newcastle upon Tyne, NE1 4LP, UK

## Abstract

**Background:**

Whilst there has been increasing research interest in interventions which promote physical activity during pregnancy few studies have yielded detailed insights into the views and experiences of overweight and obese pregnant women themselves. The qualitative study described in this paper aimed to: (i) explore the views and experiences of overweight and obese pregnant women; and (ii) inform interventions which could promote the adoption of physical activity during pregnancy.

**Methods:**

The study was framed by a combined Subtle Realism and Theory of Planned Behaviour (TPB) approach. This enabled us to examine the hypothetical pathway between beliefs and physical activity intentions within the context of day to day life. The study sample for the qualitative study was chosen by stratified, purposive sampling from a previous study of physical activity measurements in pregnancy. Research participants for the current study were recruited on the basis of Body Mass Index (BMI) at booking and parity. Semi-structured, in-depth interviews were conducted with 14 overweight and obese pregnant women. Data analysis was undertaken using a Framework Approach and was informed by TPB.

**Results:**

Healthy eating was often viewed as being of greater importance for the health of mother and baby than participation in physical activity. A commonly cited motivator for maintaining physical activity during pregnancy is an aid to reducing pregnancy-related weight gain. However, participants often described how they would wait until the postnatal period to try and lose weight. A wide range of barriers to physical activity during pregnancy were highlighted including both internal (physical and psychological) and external (work, family, time and environmental). The study participants also lacked access to consistent information, advice and support on the benefits of physical activity during pregnancy.

**Conclusions:**

Interventions to encourage recommended levels of physical activity in pregnancy should be accompanied by accessible and consistent information about the positive effects for mother and baby. More research is required to examine how to overcome barriers to physical activity and to understand which interventions could be most effective for overweight/obese pregnant women. Midwives should be encouraged to do more to promote activity in pregnancy.

## Background

An increasing proportion of women in the UK are obese or overweight at the start of their pregnancy [1,2]. This may result in adverse consequences for the immediate and longer-term health of the mother and child [3,4]. In light of this, pregnancy is emerging as a priority area for interventions which aim to address the obesity epidemic. Pregnancy is a unique and critical period in the life course for women and, consequently, they may be more receptive to behaviour change interventions [5]. Physical activity promotion is a key component of weight control interventions [6] as it has beneficial effects on glucose metabolism. It has also been suggested that this may improve pregnancy outcomes independent of weight [7]. National guidance in the UK recommends at least 30 minutes of moderate intensity activity a day throughout pregnancy for most women [8,9]. However, research has highlighted that pregnant women often have low total physical activity levels [10] and that these tend to reduce further in the later stages of pregnancy [11].

There is a dearth of information regarding women's attitudes to physical activity in pregnancy. A small number of studies have highlighted significant barriers to participation, including lack of time, lack of facilities (including childcare) and physical barriers [12-15]. Indeed it has been found that some women perceive physical activity to be an unsafe behaviour when pregnant [16,17]. However, few studies have offered detailed insights into the views and experiences of overweight and obese women themselves. This paper provides qualitative insights into how overweight and obese pregnant women living in the UK feel about physical activity in pregnancy within the context of their day to day lives. The findings highlight the barriers and motivators which surround physical activity in pregnancy, the conflicting advice and information which women receive during pregnancy, and which criteria should be considered when designing effective interventions for physical activity in pregnancy.

## Methods

### Theoretical Framework

The study was framed by a theoretical approach which combined both Subtle Realism [18] and the Theory of Planned Behaviour (TPB). This combined approach enabled an examination of the hypothetical pathway between beliefs and physical activity intentions within the context of a woman's day to day life. The Subtle Realism approach [18] stipulates that the social world does exist independently of individual subjective understandings, but is only accessible to us via research participants' interpretations [19]. The TPB is a psychological model widely used in the design of behaviour change interventions [20]. The TPB approach stipulates that intentions predict behaviour, and that three sets of beliefs mediate behavioural intentions in relation to the following criteria: (i) behavioural beliefs, i.e. attitudes based on perceived benefits and harms, (ii) control beliefs, i.e. perceptions relating to control over necessary resources and support to engage in the relevant behaviour; and (iii) normative beliefs i.e. subjective norms, determined by perceptions of the views of others. Using a combined TPB and Subtle Realism approach it was possible to examine women's views and experiences within the context of their day to day concerns and priorities.

### Study participants

Ethical approval was given by the Durham and Tees Valley 2 Rec (Ref:07/H0908/53), and written informed consent was obtained from the study participants. The sampling frame consisted of 65 women participating in a feasibility study of physical activity measurement methods. Inclusion criteria for the previous study were: (i) any woman booking with a normal, singleton pregnancy, (ii) a measured body mass index (BMI) at booking (in the first trimester) greater than or equal to 25 kg/m^2 ^(i.e. overweight or obese), (iii) adequate verbal and written English and (iv) older than 16 yrs. Of the women participating in the measurement study, around 40% were obese, 47% were nulliparous (no previous children), 93% were white, 76% were employed at recruitment and 33% were educated to degree level. These women agreed to wear an accelerometer for seven days at two or three time points during pregnancy and to complete two different physical activity questionnaires at each data collection. The measurement study was designed to compare self report and objective measurement methods in pregnant women during normal daily activities.

Previous research has highlighted that weight and family size may influence physical activity participation in non-pregnant women [21,22]. Thus, a stratified purposive sample [19] for the current study was undertaken according to BMI and parity. These criteria allowed some diversity in the sample and thus a broad range of women's experiences was explored. Of the 22 women invited to participate in the study, 14 agreed to be interviewed. All of the 8 women who declined to be interviewed, or were not contactable, were in the obese category (BMI 30 kg/m^2 ^or more). Reasons given for non-participation included chronic health problems and time constraints. The final sample of 14 interviewees included eight who were obese and eight who were in their first pregnancy. Thirteen were white, thirteen were employed and five were educated to degree level. All women were interviewed in late pregnancy.

### Data collection and analysis

Semi-structured, in-depth interviews based on a topic guide were used to enable a detailed exploration of women's views and experiences using a flexible and responsive approach [19]. Interviews were audio-recorded at the participant's home, with participant permission, and lasted between 45-55 minutes. The topic guide included the following prompts to elicit participant views and experiences: (i) what a 'healthy lifestyle' means and its relative relevance for pregnancy; (ii) physical activity in pregnancy (the benefits, barriers and influences); (iii) healthy lifestyle interventions in pregnancy and the improvements that could be made.

Data analysis was undertaken using a Framework Approach to manage, describe and explore the original data in relation to the underlying TPB [19]. The interview transcripts were indexed and mapped on the basis of recurring themes. The synthesised data were examined to identify explanatory accounts, and preliminary typologies were developed [19].

It is important that researchers reflexively examine their research as "knowledge is produced in specific circumstances and that those circumstances shape it in some way" (Rose, 1997, p.305) [23]. For the research design and analysis phases of the study there were three checks on validity (i) A topic guide was used to ensure a similar range of topics was discussed with each participant. (ii) In order to ensure the reliability of the study's coding framework two members of the research team (ZW and JB) read five of the interviews and agreed the coding framework and (iii) A topic guide was used to ensure a similar range of topics was discussed with each participant. Although only a small number of interviews were conducted, data saturation (i.e. where no new themes were emerging) was achieved after only 12 interviews and confirmed with the two final interviews.

## Results

The findings are presented according to the analytical typologies: (i) behavioural beliefs and attitudes, (ii) control beliefs and (iii) normative beliefs. Verbatim quotes from the study participants are labelled in terms of their age, whether they were overweight (BMI 25-30 kg/m^2^) or obese (BMI over 30 kg/m^2^) and whether they were nulliparous (NP) or multiparous (MP).

### (i) Behavioural beliefs/attitudes

Participants were aware of, and broadly endorsed, the importance of a 'healthy diet' and 'being physically active' in pregnancy. The antenatal period was often viewed as an opportunity to make positive lifestyle changes:

"Being pregnant perhaps is a good trigger, a good motivational point.....to actually maybe take stock of what you are doing, or what you are not doing. And to start making choices which will continue after you're pregnant." (Age 28, overweight, NP)

"This is a time when I'm thinking of health things..."(Age: 20, overweight, NP)

However, awareness of needing to adopt a healthy lifestyle during pregnancy was not always enough to initiate behavioural change:

"It's not news that you've got to eat well and not smoke and not drink and do activity in pregnancy.....I think people are aware and choose to ignore it." (Age 31, obese, MP)

"It's all personal choice at the end of the day....all the information's there and I think people just deal with the bits they want to deal with." (Age 28, overweight, NP)

A range of perceived benefits of being physically active during pregnancy were elicited. Regaining pre-pregnancy weight and body shape were the most commonly cited benefits:

"It's about myself and about wanting to be able to get back in to a healthy shape once the baby's born, and not being fat forever." (Age 20, overweight, NP)

Other perceived physical benefits of being physically active in pregnancy included an easier pregnancy and labour:

"You just generally feel healthier. You get through your pregnancy a lot better." (Age 31, obese, MP)

"The fitter you are throughout your pregnancy, the more supple you are. And you're supposed to have an easier time giving birth." (Age 30, obese, MP)

The psychological benefits of being physically active in pregnancy were also cited:

"You feel more alive, don't you? The more you do... you don't feel like a couch potato. You feel better about yourself." (Age 30, obese, MP)

"If your body feels healthier you feel healthier in your mind.....it all just makes you feel better in yourself...." (Age 33, obese, MP)

However, perceived benefits of being physically active in pregnancy were only articulated by our study participants in relation to their own health. Specific benefits associated with physical activity for the baby were not mentioned spontaneously (i.e. without prompting) by any of the study participants. Indeed some women were concerned that undertaking physical activity during pregnancy may have a harmful effect on their baby:

"I don't want to do anything that is going to harm the baby." (Age 29, obese, NP)

"It was more a case of making sure that the baby was safe and the baby was all right. That was my main worry." (Age 26, overweight, NP)

All of study participants appeared relatively unconcerned about weight gain during pregnancy. Weight gain was perceived to be a "natural" and "acceptable" part of being pregnant. For many women, any action to address pregnancy weight gain was deferred to the postnatal period:

"I take it for granted that I will put on weight." (Age 37, obese, MP)

"I think like you've got a good excuse to put on weight when you're pregnant so I haven't been that bothered." (Age 28, overweight, NP)

"I'm conscious that I'm going to try to do more after this baby is born." (Age 31, obese, MP)

Study participants also tended to feel that healthy eating was more important than being physically active in pregnancy. They often cited the baby as being the main beneficiary of a healthy diet (which was in marked contrast to their views on physical activity described earlier):

"Obviously anything you're taking in can sort of make its way to the baby and will have an effect on the growth of baby and things like that." (Age 37, obese, MP)

"It's just important that you give the baby good nutrients and good food." (Age 33, obese, MP)

On the basis of the findings, participants may be broadly classified into three groups: (i) 'diet-emphasisers,' who felt healthy eating had more important beneficial effects for both mother and baby than physical activity; (ii) 'diet/activity-equalisers' for whom both diet and physical activity were important, and (iii) 'activity-emphasisers' who physical activity was more important than diet. Only one participant was classified as an 'activity-emphasiser; she acknowledged the importance of healthy eating but felt physical activity could act as an important 'compensator' to burn off excess intake.

### (ii) Control beliefs

The study participants cited several perceived internal and external barriers to physical activity in pregnancy. Personal health problems associated with pregnancy were the most commonly cited 'internal' barrier to physical activity. Such barriers included sickness, lack of energy, and feeling uncomfortable due to size:

"I feel kind of, I suppose, at the mercy of what my body is going to do to me. If I feel exhausted I just kind of go with it really, rather than push myself." (Age 34, overweight, MP)

"You just get heavier and you are bigger and it is harder to move and everything is more uncomfortable." (Age 29, obese, NP)

Other internal barriers associated with physical activity in pregnancy included lack of self-confidence and motivation:

"With me being overweight, I'm just too paranoid to go swimming." (Age 30, obese, MP)

"I think some of it is just sheer laziness.... and routine that stopped me taking that opportunity [to be physically active]." (Age 21, obese, NP)

Work was the most commonly cited external barrier to physical activity. It was perceived to have a negative impact on available time and energy levels, the ability to commit to a regular exercise class, and also meant having to prioritise in relation to other day to day life activities:

"Because of the pressures of work...I didn't have time during the working week to do it." (Age 28, overweight, NP)

Study participants with children often cited barriers to physical activity which included lack of time, lack of suitable childcare, guilt, and wanting to spend time with their family:

"It's difficult with children to fit extra things in." (Age 30, obese, MP)

"I would rather spend time with my daughter rather than go off and do a swim because I would feel a bit guilty...." (Age 34, overweight, MP)

"I personally would just prefer to look after [name of child].... I don't see it as a great sacrifice" (Age 33, obese, MP)

Some women felt that there was a lack of suitable exercise classes for pregnant women:

"If there were more activities offered I would probably do more activities." (Age 29, obese, NP)

"There's a lot of social things but as far as physical stuff for mums...I don't think there's a huge amount offered." (Age 31, obese, MP)

"I know if there was something out there I would use it.....and if there is anything out there, I don't know about it.....it's not publicised." (Age 29, obese, NP)

Other perceived barriers to physical activity mentioned by the study participants included not feeling safe when they were "out and about" in their local neighbourhood (especially at nights), the weather, and financial constraints.

### (iii) Normative beliefs

The study participants often highlighted how they felt they had not had adequate levels of information, support, or advice regarding physical activity in pregnancy:

"Other than my midwife just saying, you know, just carry on as normal...(there was) nothing specific from any health professionals." (Age 31, obese, MP)

"I might be doing the midwife, who's lovely, a total injustice. But there's nothing that's standing out in my head that I can think of at the moment that's provided me with that sort of information or, or that kind of guidance about, about lifestyle or exercise or diet or anything." (Age 28, overweight, NP)

Although study participants felt that all health professionals had a responsibility to provide advice and guidance about the benefits or resources available, they generally felt that the midwife was the most appropriate person to provide support and guidance. Typologies developed around the perceived behavioural expectations of midwives emphasised how most of the study participants felt that their midwife had assumed a 'non-advisory' role, i.e. they had provided minimal or no activity advice during pregnancy. No midwives had been perceived to offer an 'active advisory' role, i.e. where they would actively encourage women to be physically active during pregnancy.

Whilst study participants had lacked guidance on physical activity during pregnancy from health professionals they had often been the recipients of lay knowledge and expertise from family members and partners. Often such advice was conflicting. For example, some participants had described how their mothers had actively discouraged physical activity, whereas partners were more likely to be sources of encouragement:

"My mum has been oh, give up work, cut work down and that kind of thing...and you know, stop the walking, stop going out with the dogs, stop doing this, stop doing that..." (Age 26, overweight, NP)

"He (husband) is like that,' get up woman, you're just pregnant, you're not ill."' (Age 29, obese, NP)

Most of the study participants had accessed some form of media-based pregnancy information during their pregnancy (e.g. via TV programmes and pregnancy related websites such as netmums). Some had also read pregnancy books. However, the lifestyle in pregnancy information which had been accessed by the study participants was perceived to be negative, conflicting and impersonal:

"...its always you shouldn't do this, this and this.... if you listened and took notice of everything that you heard, you wouldn't know where you were..." (Age 34, overweight, MP)

"You read so much or you get so much off the internet or whatever that it can be quite overwhelming. And the information is conflicting..." (Age 28, overweight, NP)

## Discussion

This paper has explored views and experiences of physical activity during pregnancy among overweight and obese women. By adopting a combined Subtle Realism and TPB approach we have been able to highlight a range of behavioural, control and normative beliefs which impact on women's intentions to being physically active in pregnancy. These views and experiences should be taken into account when designing interventions to promote increased physical activity during pregnancy.

In interpreting the findings of this study, it is important to acknowledge its limitations. The sample size was small and derived from women who had already participated in a study of physical activity in pregnancy and thus may have already been motivated to maintain a healthy lifestyle. The participants were also relatively well educated with a high level of employment. The findings may therefore not be generalisable to other settings or to women from black and minority ethnic groups (since there was only one non-white participant in the sample).

In order to strive for objectivity and neutrality in research it is important to reflect on how bias may creep into the qualitative research and thus threaten validity [19]. In particular, it is essential to reflect on how the interviewer was "placed" by participants [23]. The interviewer for this study was a health professional, and someone who was pregnant during the interviews. Whilst the interviewer distanced herself from the role of the 'expert' at the start of each interview, and stressed she was only interested in participant's own beliefs and attitudes (stressing there were no right or wrong answers), participant 'placing' may still have influenced the interview dialogue.

In this study validity was maximised by a topic guide being used and two researchers deriving a thematic framework from the data (which provided a reliable and trustworthy context for data interpretation). A number of further steps were taken to enhance the validity of the study. A topic guide was used, and interviewing continued until data saturation was achieved (although achieving data saturation after only 12 interviews is likely to have been influenced by the homogenous study sample). Also, many of the themes which arise from the study are similar to those reported from studies undertaken in other settings. This gives further confidence in the reliability of the findings.

The main perceived benefits of being physically active during pregnancy highlighted by this study, were (1) to minimise pregnancy-related weight gain and (2) facilitate a return to pre-pregnancy body weight and shape. However, beliefs about the potential harm to the baby from physical activity were also evident and specific benefits to the baby were not elicited by the research participants without prompting by the interviewer. The findings also suggest that whilst study participants often advocated the importance of maintaining healthy eating and activity behaviours during pregnancy, healthy eating was generally perceived to be of greater importance, particularly for the baby's health, than physical activity. These perceptions are likely to be influenced by the information sources that women have access to in pregnancy and the conflicting messages contained within them [24]. Participants were often positively motivated towards eating healthily due to the perceived positive effects of a healthy diet on their baby's health and growth. Other studies have also identified the baby as a primary motivator for enhancing nutrition awareness in pregnancy [25,26]. Interventions to promote recommended levels of physical activity in pregnancy need to provide specific information on fetal safety and also stress the potential benefits to the mother.

It is notable that most of the study participants who were interviewed were unconcerned about weight gain during pregnancy. They tended to defer any intention to address pregnancy-related weight gain to the postnatal period. This attitude may reflect the current absence of formal recommendations for pregnancy weight gain in the UK. This is in direct contrast to the US [27]. Whilst there is strong epidemiological evidence which suggests excessive pregnancy weight gain is associated with adverse outcomes for mother and infant [28], there is only limited evidence that weight management interventions in pregnancy are effective [29,30]. Thus, there is a need to develop and evaluate new approaches to diet and activity interventions in pregnancy. And in particular, to establish the relative importance of dietary composition and physical activity levels, as well as weight gain, in determining pregnancy outcome.

In relation to control beliefs, study participants highlighted a large number of barriers to participating in physical activity in pregnancy. These included both internal (physical and psychological) and external (work, family, time and environmental) barriers. These findings are broadly consistent with other studies [13-17]. Since many of these barriers were perceived to be outside of a women's control, any behaviour change interventions need to support women to find solutions which effectively overcome these barriers. One approach may be to emphasise practical and feasible strategies, such as building in an activity like walking, into a pregnant women's everyday life routines. Encouraging physical activity, particularly during the second trimester (when nausea and fatigue have diminished) and women are not constrained by physical size, may also be effective.

The exploration of normative beliefs arising from the findings suggests women are influenced by the attitudes of partners, family members and by sources of information in the media, books and website. Partners were cited as having positive influences in maintaining activity, in contrast to other family members.

One of the most important issues arising from this study was the perceived lack of accessible information and advice on the benefits of physical activity during pregnancy. Midwives were viewed as being ideally placed to advise and support women about physical activity in pregnancy. However, many of the study participants described how their midwives had not given them any advice or guidance on physical activity. It has also been suggested from the findings of previous research that healthcare providers are often reluctant to advise on diet and activity changes during pregnancy [31]. Little is known about why this is the case. Other research has highlighted how midwives often use language to direct mothers to making the best decision around baby feeding [32]. This contrasts sharply with the findings presented in this paper. The passing on of 'superior knowledge' does not appear to take place in relation to physical activity. Whilst it must be acknowledged that the current workload of midwives is "excessive" due to a shortage of midwives,[33] there is a potential role for midwives in offering advice and support. And interventions to promote activity in pregnancy should involve them. The findings also emphasise the potential of using the media as a vehicle by which to communicate health information in pregnancy [28]. However, doing this effectively is challenging.

## Conclusions

A healthy lifestyle incorporating recommended levels of physical activity during pregnancy may contribute to improved pregnancy outcomes. By using a combined Subtle Realism and TPB approach this study has yielded detailed insights into a number of behavioural, control and normative beliefs which influence women's behavioural intentions. Such insights could be targeted by interventions to promote physical activity in pregnancy. The findings suggest little awareness of the potential benefits of physical activity for the baby. The study also highlights the significance of perceived barriers to participation in physical activity. A focus on benefits for the baby, and on facilitating return to pre-pregnancy weight and appearance, are specific messages that may motivate women to be more active during pregnancy. Also provision of personalised support by the midwife is lacking. Interventions which harness the media, or involve partners, are potential approaches to consider for a proposed intervention in pregnancy. Extending interventions to the postnatal period may also prove fruitful [31].

## Competing interests

The authors declare that they have no competing interests.

## Authors' contributions

ZW undertook the interviews and analysis and wrote the first draft of the manuscript. JB contributed to the analysis and writing of the manuscript and took the lead in redrafting the script following editorial review. SCR contributed to the conception and design of the study and reviewed the manuscript. CMcP recruited the study participants and reviewed the manuscript. JR contributed to the design of the study and reviewed the manuscript. RB contributed to conception and design of the study and writing of the manuscript. All of the authors have read and approved the final manuscript.

## Pre-publication history

The pre-publication history for this paper can be accessed here:

http://www.biomedcentral.com/1471-2393/10/18/prepub
